# Volasertib Enhances Sensitivity to TRAIL in Renal Carcinoma Caki Cells through Downregulation of c-FLIP Expression

**DOI:** 10.3390/ijms18122568

**Published:** 2017-11-29

**Authors:** Mi-Yeon Jeon, Kyoung-jin Min, Seon Min Woo, Seung Un Seo, Shin Kim, Jong-Wook Park, Taeg Kyu Kwon

**Affiliations:** Department of Immunology, School of Mediine, Keimyung University, 2800 Dalgubeoldaero, Dalseo-Gu, Daegu 704-701, Korea; dldkfls2333@naver.com (M.-Y.J.); kyoungjin.min@gmail.com (K.-j.M.); woosm724@gmail.com (S.M.W.); sbr2010@hanmail.net (S.U.S.); god98005@dsmc.or.kr (S.K.); j303nih@dsmc.or.kr (J.-W.P.)

**Keywords:** volasertib, TRAIL, c-FLIP, caspase, apoptosis

## Abstract

Polo-like kinase 1 (PLK1) plays major roles in cell cycle control and DNA damage response. Therefore, PLK1 has been investigated as a target for cancer therapy. Volasertib is the second-in class dihydropteridinone derivate that is a specific PLK1 inhibitor. In this study, we examined that combining PLK1 inhibitor with tumor necrosis factor-related apoptosis-inducing ligand (TRAIL) would have an additive and synergistic effect on induction of apoptosis in cancer cells. We found that volasertib alone and TRAIL alone had no effect on apoptosis, but the combined treatment of volasertib and TRAIL markedly induced apoptosis in Caki (renal carcinoma), A498 (renal carcinoma) and A549 (lung carcinoma) cells, but not in normal cells (human skin fibroblast cells and mesangial cells). Combined treatment induced accumulation of sub-G1 phase, DNA fragmentation, cleavage of poly (ADP-ribose) polymerase (PARP) and activation of caspase 3 activity in Caki cells. Interestingly, combined treatment induced downregulation of cellular-FLICE-inhibitory protein (c-FLIP) expression and ectopic expression of c-FLIP markedly blocked combined treatment-induced apoptosis. Therefore, this study demonstrates that volasertib may sensitize TRAIL-induced apoptosis in Caki cells via downregulation of c-FLIP.

## 1. Introduction

Recently, tumor necrosis factor-related apoptosis-inducing ligand (TRAIL) has been shown to induce apoptosis in a multiple of cancer cells, but TRAIL has no effect on the apoptosis of normal cells [1,2]. However, treatment with TRAIL may be insufficient for cancer therapy because some cancer cells are resistant to the apoptotic effects of TRAIL [3,4]. TRAIL-resistant cancer cells can be sensitized by cancer chemopreventive agents, suggesting that combined treatment may be a possibility. Therefore, identification of TRAIL sensitizers is required to overcome TRAIL resistance. 

Polo-like kinase 1 (PLK1) is a serine/threonine kinase that plays a key role in G2/M transition through cytokinesis [5]. Elevated levels of PLK1 have been reported in multiple cancer types, including melanoma, prostate, breast, colorectal, and renal cancer [6,7]. The overexpression of PLK1 has been linked to decreased survival rate and poor prognosis in various cancer types [6,8]. Volasertib (BI 6727) is a PLK1 inhibitor [9] and has been reported as a potential therapeutic agent in multiple cancer types. Recently, PLK1 inhibitor-resistant cell lines have been reported [10]. To improve the clinical benefits of PLK inhibitors, combinations of anticancer therapies with high efficacy and low toxicities are highly sought after. However, whether volasertib has sensitizing effects against TRAIL-mediated apoptotic cell death remains unclear. 

In this present study, we show that volasertib can significantly enhance TRAIL-induced apoptosis in various cancer cells. Furthermore, we show here for the first time that downregulation of c-FLIP expression is a major role in sensitizing effect of volasertib on TRAIL-induced apoptosis in cancer cells. 

## 2. Results

### 2.1. The Effect of Volasertib (BI 6727), a Polo-Like Kinase1 (PLK1) Inhibitor, on Apoptotic Cell Death in Human Renal Carcinoma Caki Cells

**Figure 1:**
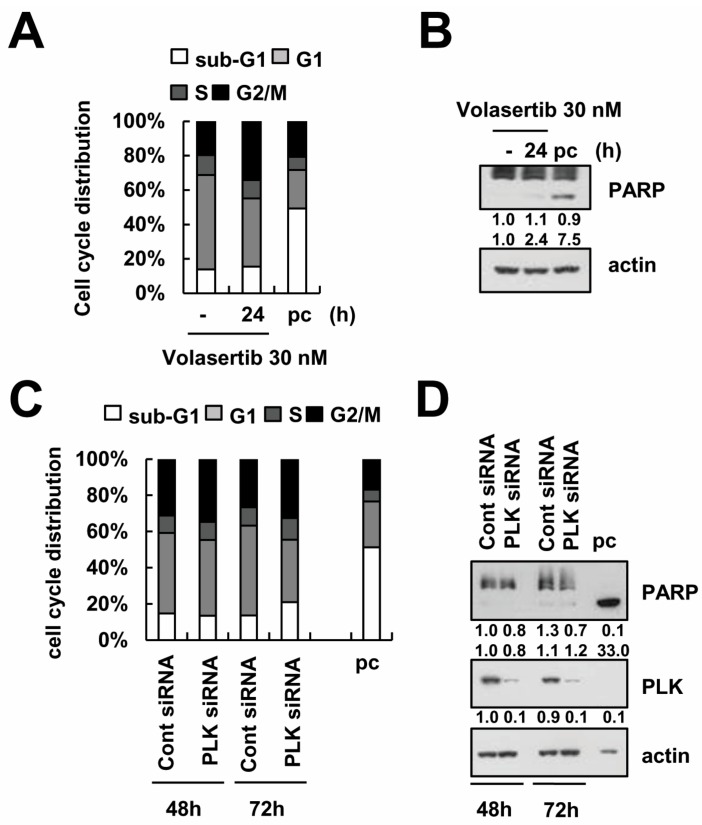
The effect of volasertib on cell cycle distribution and apoptosis in Caki cells. (**A**,**B**) Caki cells were treated with 30 nM volasertib for 24 h. The DNA content was analyzed by flow cytometer (**A**). The expression of PARP and actin were determined via Western blotting (**B**). (**C**,**D**) Caki cells were transiently transfected with control siRNA (Cont siRNA) or PLK siRNA for the indicated time periods. After transfection, the DNA content was analyzed with a flow cytometer (**C**). The protein levels of PARP, PLK, and actin were determined via Western blotting. The level of actin was used as a loading control (**D**). P.C.: positive control (10 ng/mL TNF-α plus 5 μg/mL cycloheximide for 14 h).

Volasertib has been shown to cause cell cycle arrest and to induce apoptosis in various cancer cells at nanomolar concentrations [11,12]. To investigate whether PLK1 inhibition induces apoptosis and cell cycle arrest in human renal carcinoma Caki cells, we assessed cell cycle, the apoptotic hypo-diploid sub-G1 phase, and the cleavage of PARP. Interestingly, 30 nM volasertib did not induce a significant change in the sub-G1 phase or the cleavage of PARP (Figure 1A,B). However, volasertib increased the population of the G2/M phase (Figure 1A). In addition, downregulation of PLK1 by siRNA also had not induced apoptosis or PARP cleavage after 72 h (Figure 1C,D). These results suggest that Caki cells are resistant to volasertib treatment. Therefore, we studied the effect of this PLK1 inhibitor on Caki cells as a single agent and in combination with TRAIL.

### 2.2. The Effect of Volasertib on TRAIL-Induced Apoptotic Cell Death in Human Renal Carcinoma Caki Cells

**Figure 2:**
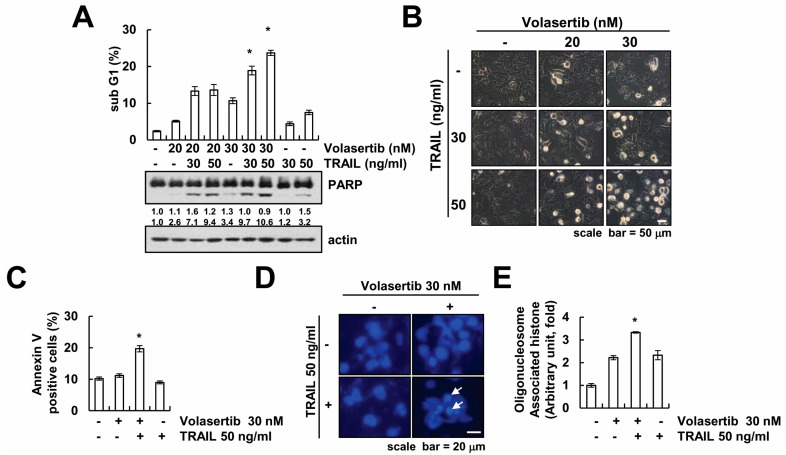
Volasertib sensitizes TRAIL-induced apoptosis in Caki cells. (**A**,**B**) Caki cells were treated with TRAIL (30 or 50 ng/mL) in the presence or absence of volasertib (20 or 30 nM) for 24 h. The sub-G1 fraction was detected via flow cytometry. The expression of PARP and actin were determined via Western blotting (**A**). The cell morphology was examined using interference light microscopy (**B**). (**C**–**E**) Caki cells were treated with 50 ng/mL TRAIL in the presence or absence of 30 nM volasertib for 24 h. Annexin V positive cells were analyzed via flow cytometry (**C**). The nuclei and fragmented DNA were detected by 4′,6′-diamidino-2-phenylindole staining (**D**) and DNA fragmentation detection kit (**E**). The white arrows indicated the condensation of chromatin and nuclear fragmentation. The values in graphs (**A**,**C**,**E**) represent the mean ± standard deviation (SD) from three independent samples. * *p* < 0.01 compared to the control.

To investigate whether volasertib enhance TRAIL-induced apoptotic cell death, Caki cells were treated with volasertib alone (20, 30 nM), TRAIL alone (30, 50 ng/mL), and with volasertib and TRAIL combined. Apoptotic cell death was assayed via flow cytometry analysis and Western blotting. The combined treatment of volasertib and TRAIL markedly induced an accumulation of apoptotic hypo-diploid sub-G1 phase and cleavage of PARP in a dose-dependent manner (Figure 2A). However, neither volasertib alone nor TRAIL alone induced apoptotic cell death. The combined treatment of volasertib and TRAIL caused apoptotic morphology such as apoptotic body formation, cell shrinkage, and cell detachment on the plate (Figure 2B). Furthermore, the combined treatment of volasertib and TRAIL induced annexin V positive cells (Figure 2C). In addition, we tested nuclear condensation and DNA fragmentation, which is the hallmark of apoptosis. 4′,6′-diamidino-2-phenylindole (DAPI) staining and a cytoplasmic histone-associated DNA fragment assay showed nuclear condensation and DNA fragmentation in the combined treatment of volasertib and TRAIL (Figure 2D,E).

### 2.3. Volasertib-Induced Apoptosis Is Caspase-Dependent in Caki Cells

**Figure 3:**
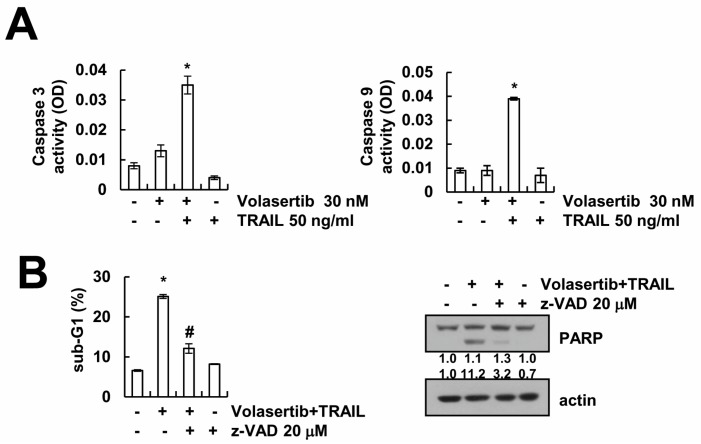
The combined treatment of volasertib and TRAIL induces caspase-mediated apoptosis in Caki cells. (**A**) Caki cells were treated with 30 nM volasertib plus 50 ng/mL TRAIL for 24 h. Caspase activities was determined with colorimetric assays using caspase-3 DEVDase or caspase-9 LEHDase assay kits. (**B**) Caki cells were treated with 30 nM volasertib plus 50 ng/mL TRAIL in the presence or absence of 20 μM z-VAD-fmk (z-VAD) for 24 h. The sub-G1 fraction was detected via flow cytometry. The expression of PARP and actin were determined via Western blotting. The values in graphs (**A**,**B**) represent the mean ± SD from three independent samples. * *p* < 0.01 compared to the control. ^#^ *p* < 0.01 compared to volasertib plus TRAIL.

Next, we determined whether volasertib plus TRAIL-induced apoptosis is associated with the activation of the caspase-3. We had already found that the combined treatment of volasertib and TRAIL induced the cleavage of PARP, which is one of the substrates of activated caspase-3 (Figure 2A). Combined treatment increased caspase-3 activity (Figure 3A). To confirm the roles of caspase-3 activation in the volasertib plus TRAIL-induced apoptosis, we performed pan-caspase inhibitor assay. As shown in Figure 3B, treatment with z-VAD-fmk, a pan-caspase inhibitor, inhibited the induction of sub-G1 population and cleavage of PARP. These finding suggested that the combined treatment of volasertib plus TRAIL-induced apoptosis is associated with caspase-3 activation.

### 2.4. Combined Treatment Volasertib and TRAIL Induces the Downregulation of c-FLIP Expression 

**Figure 4:**
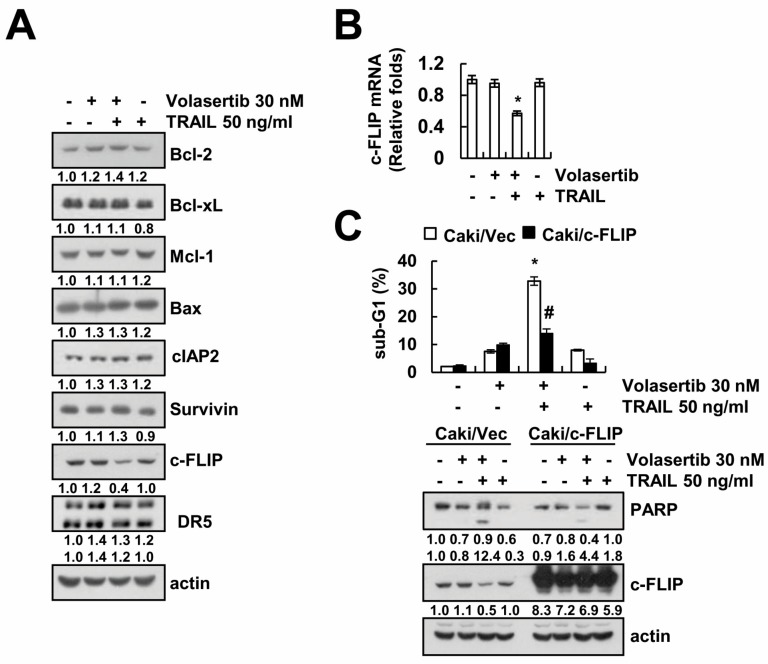
The downregulation of c-FLIP is associated with the induction of combined treatment-induced apoptosis. (**A**,**B**) Caki cells were treated with 50 ng/mL TRAIL in the presence or absence of 30 nM volasertib for 24 h. The protein expression levels of Bcl-2, Bcl-xL, Mcl-1, Bax, cIAP2, survivin, c-FLIP, DR5, and actin were determined via Western blotting (**A**). The mRNA expression levels of c-FLIP and actin were determined by qPCR (**B**). (**C**) Cells (Caki/Vec and Caki/c-FLIP) were treated with 50 ng/mL TRAIL in the presence or absence of 30 nM volasertib for 24 h. The sub-G1 fraction was detected via flow cytometry. The protein expression levels of PARP, c-FLIP, and actin were determined via Western blotting. The values in graphs (**B**,**C**) represent the mean ± SD from three independent samples. * *p* < 0.01 compared to the control. ^#^ *p* < 0.01 compared to volasertib plus TRAIL-treated Caki/Vec.

To determine whether apoptosis-related proteins are involved in the combined treatment of volasertib and TRAIL, we measured the expression levels of apoptosis-related proteins. Combined treatment markedly induced downregulation of c-FLIP expression, whereas expression of apoptosis related proteins (Bcl-2, Bcl-xL, Mcl-1, Bax, cIAP2, DR5, and survivin) did not change (Figure 4A). Next, we investigated whether the combined treatment of volasertib and TRAIL induces the downregulation of c-FLIP expression at the transcriptional levels. As shown in Figure 4B, combined treatment induced downregulation of c-FLIP mRNA expression. To investigate the role of the downregulation of c-FLIP protein in combined treatment-induced apoptosis, we used c-FLIP-overexpressing cells. Overexpression of c-FLIP attenuated combined treatment-induced apoptosis and PARP cleavage (Figure 4C). These results suggest that the downregulation of c-FLIP expression is an important role in the combined treatment of volasertib and TRAIL-induced apoptosis.

### 2.5. Volasertib-Mediated TRAIL Sensitization Is Not Associated with Induction of ER Stress and ROS Signaling Pathway

**Figure 5:**
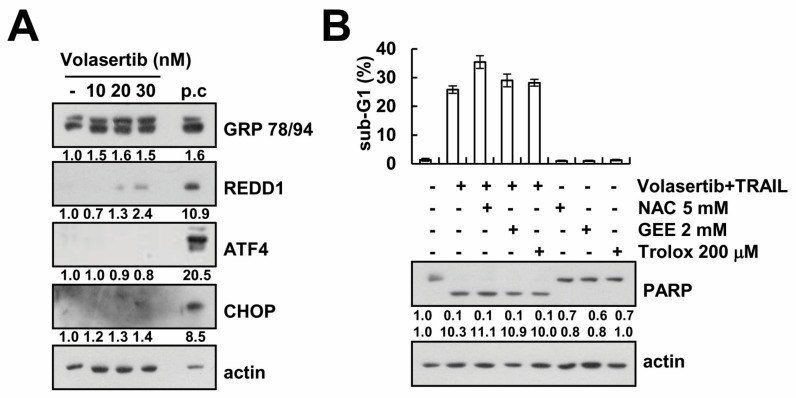
TRAIL sensitization by volasertib is independent of ER stress and ROS signaling pathway. (**A**) Caki cells were treated with the indicated concentrations of volasertib or 2 μM brefeldin A (P.C; positive control) for 6 h. The protein expression levels of Grp78/94, REDD1, ATF4, CHOP, and actin were determined via Western blotting. (**B**) Caki cells were pretreated with 5 mM NAC, 2 mM GEE, and 200 μM trolox for 30 min, and then supplemented with 50 ng/mL TRAIL plus 30 nM volasertib for 24 h. The sub-G1 fraction was measured via flow cytometry. The expression of PARP and actin were determined via Western blotting. The values in graph (**B**) represent the mean ± SD from three independent samples.

Next, we investigated whether volasertib induces endoplasmic reticulum (ER) stress. Western blotting analysis showed that Grp78/94 was upregulated by volasertib in a dose-dependent manner. However, the key transcriptional factors expression of ER stress (ATF4, CHOP, and REDD1) was not altered (ATF4 and CHOP) or slightly increased (REDD1) in response to volasertib (Figure 5A). In addition, to investigate the role of reactive oxygen species (ROS) in the combined treatment of volasertib and TRAIL, we analyzed cell death in the presence and absence of ROS scavengers (NAC, GEE, and trolox). ROS scavengers failed to rescue Caki cells from volasertib plus TRAIL-induced apoptosis (Figure 5B). Therefore, volasertib-mediated TRAIL sensitization is independent of ER stress and ROS signaling. 

### 2.6. Knockdown of PLK1 Enhances Caki Cells to TRAIL-Mediated Apoptosis

**Figure 6:**
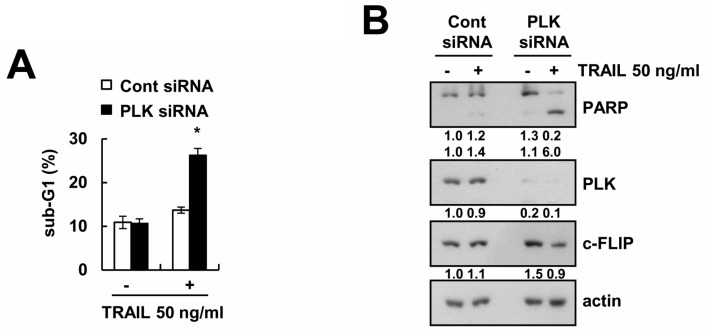
Downregulation of PLK by siRNA sensitizes TRAIL-induced apoptosis in Caki cells. (**A**,**B**) Caki cells were transiently transfected with control siRNA (Cont siRNA) or PLK siRNA. After transfection, Caki cells were treated with 50 ng/mL TRAIL for 24 h. The level of apoptosis was analyzed by the sub-G1 population using flow cytometry (**A**). The protein expression levels of PARP, PLK, c-FLIP, and actin were determined via Western blotting (**B**). The values in graph (**A**) represent the mean ± SD from three independent samples. * *p* < 0.01 compared to TRAIL-treated control siRNA.

We investigated whether the effect of volasertib on TRAIL sensitization is dependent on the inhibition of PLK1. Downregulation of PLK1 by siRNA induced apoptosis and PARP cleavage in TRAIL-treated cells (Figure 6A). Knockdown of PLK1 induced c-FLIP downregulation in TRAIL-treated cells (Figure 6B). These results suggested that both pharmacological and genetic inhibition of PLK1 can enhance Caki cells to TRAIL-induced apoptosis.

### 2.7. The Combined Treatment of Volasertib and TRAIL Induces Apoptosis in Other Cancer Cells, but Not Normal Cells

**Figure 7:**
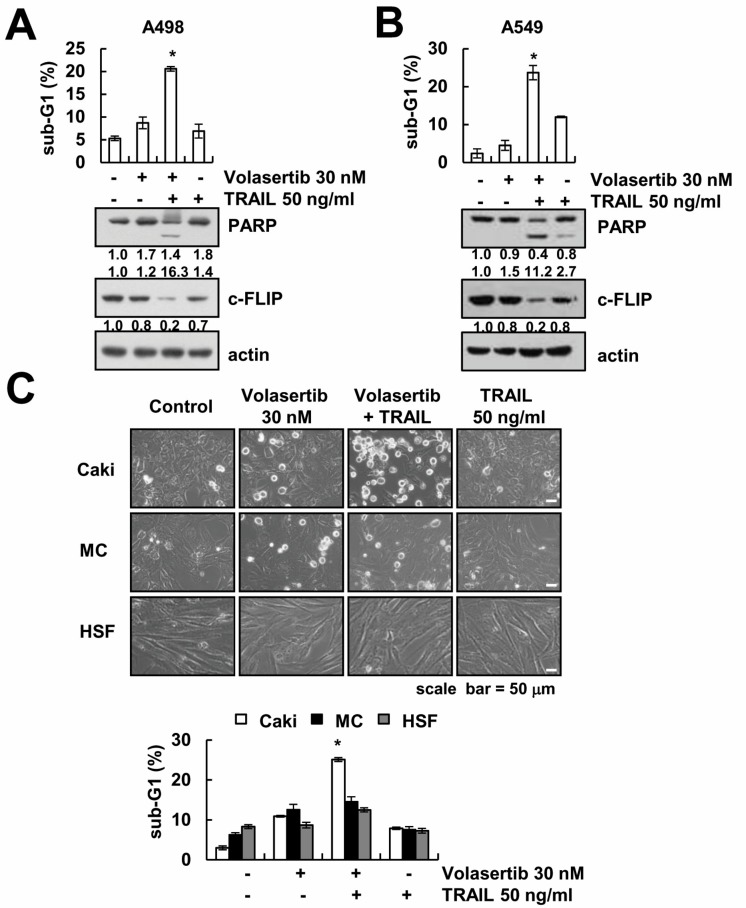
The effects of the combined treatment of volasertib and TRAIL on apoptosis in other carcinoma and normal cells. (**A**,**B**) A498 (renal carcinoma) and A549 (lung carcinoma) cells were treated with 50 ng/mL TRAIL in the presence or absence of 30 nM volasertib for 24 h. The level of apoptosis was measured by the sub-G1 fraction using flow cytometry. The protein expression levels of PARP, c-FLIP, and actin were determined via Western blotting. (**B**) Caki, mesangial cells (MC), and human skin fibroblast (HSF) cells were treated with 50 ng/mL TRAIL in the presence or absence of 30 nM volasertib for 24 h. The cell morphology was examined using interference light microscopy. The level of apoptosis was measured by the sub-G1 fraction using flow cytometry. The values in graphs (**A**–**C**) represent the mean ± SD from three independent samples. * *p* < 0.01 compared to control.

Next, we examined whether combined treatment could enhance apoptosis in other types of cancer cells and normal cells. The combined treatment of volasertib and TRAIL enhanced the sub-G1 population, PARP cleavage, and downregulation of c-FLIP expression in A498 (renal carcinoma cells) and A549 cells (lung carcinoma) (Figure 7A,B). However, volasertib plus TRAIL had no effect on morphological changes and apoptotic cell death in human skin fibroblast (HSF) cells and mesangial cells (MC) (Figure 7C). These findings indicate that volasertib enhances TRAIL-mediated apoptosis in other cancer cells, but not in normal cells.

## 3. Discussion

The main objective of this study was to demonstrate the mechanisms underlying the combined treatment of volasertib and TRAIL-induced apoptotic cell death in human renal carcinoma Caki cells. Combined treatment induced downregulation of anti-apoptotic c-FLIP protein. Human skin fibroblast (HSF) cells and mesangial cells (MC) were unaffected by combined treatment, while renal carcinoma (Caki and A498) and lung carcinoma (A549) induced apoptotic cell death by combined treatment. These data suggest that PLK1 inhibitor volasertib could be an effective TRAIL sensitizer, and combining PLK1 inhibitor with TRAIL may be an effective treatment strategy against renal cancer. 

Dihydropteridinone derivative, volasertib, acts as PLK1 inhibitor by inhibiting the enzyme activity of PLK1 in an ATP-competitive way. It is a potent and selective PLK inhibitor [9]. The inhibition of PLK1 has been shown to cause cell cycle arrest at the G2/M phase and to increase apoptosis in cancer cells [9,11,12]. Recently, Nonomiya et al. reported that the upregulation of p-glycoprotein and AKT3 and the downregulation of Myc contributed to the resistance of several PLK inhibitors [10]. The resistance mechanisms of PLK inhibitors are a critical issue to improve the clinical benefits of said PLK inhibitors. In our study, volasertib (30 nM) treatment or knockdown of PLK1 by siRNA did not induce apoptotic cell death at 72 h in Caki cells. However, the combined treatment of volasertib (30 nM) and TRAIL (50 ng/mL) caused apoptotic cell death in volasetib-resistant Caki cells, but not normal cells. 

One of the mechanisms of volasertib-mediated TRAIL sensitization is the downregulation of c-FLIP expression. Anti-apoptotic c-FLIP protein was transcriptionally regulated by the nuclear factor-κB and c-Fos pathways [13,14,15]. It was also regulated by ubiquitin- and caspase-mediated degradation [16,17,18,19]. Recently, our group reported that co-treatment with thioridazine and curcumin induced downregulation of c-FLIP through ROS production [20]. Inhibition of cathepsin S also induced downregulation of c-FLIP expression through mitochondrial ROS-mediated upregulation of Cbl expression [21]. ROS are important signaling molecules in post-translational modulation of c-FLIP protein expression [20,21]. However, in our system, ROS scavengers did not prevent the combined treatment of volasertib plus TRAIL-induced apoptosis (Figure 5B). Therefore, we need further experiments to identify the mechanism of c-FLIP downregulation in the combined treatment of volasertib and TRAIL. Ectopic expression of c-FLIP markedly blocked the combined treatment of volasertib plus TRAIL-induced apoptosis. Elevated expression of c-FLIP has been observed in several types of human cancer and has been proven to be one of the major determinants of the resistance to cell death receptor-mediated apoptosis [22,23,24,25]. 

Collectively, our results showed that volasertib enhanced TRAIL-induced apoptosis in various cancer cells, but not in normal cells. Furthermore, we demonstrated that the synergistic action of volasertib and TRAIL in human renal Caki cells is associated with the downregulation of c-FLIP expression. Therefore, our data suggest that volasertib may be effectively used as a sensitizer of TRAIL.

## 4. Materials and Methods

### 4.1. Cell Cultures and Materials

Human renal carcinoma cells (Caki and A498) and human lung carcinoma cells (A549) were obtained from the American Type Culture Collection (Manassas, VA, USA). The normal human skin fibroblasts (HSF) cells were purchased form Korea Cell Line Bank (Seoul, Korea). The primary culture of human mesangial cells (Cryo NHMC) was purchased from Clonetics (San Diego, CA, USA). The culture medium used throughout these experiments was Dulbecco’s modified Eagle’s medium (DMEM) containing 10% fetal bovine serum (FBS), 20 mM HEPES buffer, and 100 μg/mL gentamicin. Volasertib was purchased from Selleckchem (Houston, TX, USA). Recombinant human TRAIL and z-VAD-fmk was purchased from R&D (Minneapolis, MN, USA). The anti-PLK1, anti-Mcl-1, anti-Bcl-2, anti-Bcl-xL, anti-survivin, anti-cIAP2, anti-CHOP, and anti-ATF4 antibodies were purchased from Santa Cruz Biotechnology (Santa Cruz, CA, USA). The anti-Grp78 antibody was purchased from Enzo Life Sciences (Ann Arbor, MI, USA). The anti-c-FLIP antibody was obtained from ALEXIS Corporation (San Diego, CA, USA). The anti-Bax antibody was purchased from BD Biosciences (San Jose, CA, USA). The anti-PARP and anti-DR5 antibodies were purchased from Cell Signaling Technology (Beverly, MA, USA). Anti-REDD1 was obtained from Proteintech Group (Chicago, IL, USA). The anti-actin antibody was obtained from Sigma-Aldrich (St. Louis, MO, USA). Other reagents were purchased from Sigma Chemical Co. (St. Louis, MO, USA).

### 4.2. Flow Cytometry Analysis

Flow cytometry analysis was performed as described in our previous studies [20]. Briefly, the cells were suspended in 100 μL of phosphate-buffered saline (PBS), and 200 μL of 95% ethanol was added. The cells were then incubated at 4 °C for 1 h, and cells were centrifuged at 500× *g* for 5 min. After being centrifuge, cells were washed with PBS, and then resuspended in 250 μL of 1.12% sodium citrate buffer (pH 8.4) with 12.5 μg of RNase for 30 min at 37 °C. The DNA was then stained with a propidium iodide solution (50 μg/mL), and cells were analyzed via fluorescent-activated cell sorting (FACS) on a FACScan flow cytometer. 

### 4.3. Small Interfering RNA (SIRNA) 

The PLK siRNA used in this study was purchased from Santa Cruz Biotechnology (Dallas, TX, USA). The GFP (control) siRNA was purchased from Bioneer (Daejeon, Korea). Cells were transfected with siRNA oligonucleotides using Lipofectamine^®^ RNAiMAX Reagent (Invitrogen, Carlsbad, CA, USA) according to the manufacturer’s recommendations.

### 4.4. Western Blotting Analysis

Cells were washed with cold PBS and lysed on ice in 50 μL of lysis buffer (50 mM Tris–HCl, 1 mM EGTA, 1% Triton X-100, 1 mM phenylmethylsulfonyl fluoride, pH 7.5) [26,27]. Lysates were centrifuged at 10,000× *g* for 15 min at 4 °C, and the supernatant fractions were collected. Proteins were separated by SDS–PAGE and transferred to an Immobilon-P membrane. Specific proteins were detected using an enhanced chemiluminescence (ECL) Western blotting kit according to the manufacturer’s instructions.

### 4.5. Annexin V Staining

After treatment, cells were washed with PBS, and then fixed in 75% ethanol for 1 h at 4 °C. For annexin V staining, the cells were washed with ice cold PBS, and 5 μL of the annexin V conjugate was then added to each 100 μL of cell suspension. After 15 min, 400 μL of annexin V-binding buffer was used. Flow cytometry analyses were performed with a FACScan flow cytometer.

### 4.6. 4′,6′-Diamidino-2-Phenylindole Staining (DAPI) for Nuclei Condensation and Fragmentation

DAPI staining was performed as described in our previous studies [20]. The cells were fixed with 1% paraformaldehyde for 30 min at room temperature and then washed with PBS twice. The nuclei were stained with a 300 nM 4′,6′-diamidino-2-phenylindole solution (Roche, Mannheim, Germany) for 5 min, and the cells were examined by fluorescence microscopy.

### 4.7. Cell Death Assessment by DNA Fragmentation Assay 

The cell death detection ELISA plus kit (Boehringer Mannheim, Indianapolis, IN, USA) was used for assessing apoptotic activity by detecting fragmented DNA in volasertib alone, TRAIL alone, and a combination of volasertib- and TRAIL-treated cells. Briefly, each culture plate was centrifuged for 10 min at 200× *g*, the supernatant was removed, and the pellet was lysed for 30 min. After the plate was centrifuged again at 200× *g* for 10 min, and the supernatant that contained the cytoplasmic histone-associated DNA fragments was collected and incubated with an immobilized anti-histone antibody. The reaction products were incubated with a peroxidase substrate for 5 min and measured by spectrophotometry at 405 and 490 nm (reference wavelength) with a microplate reader. The signals in the wells containing the substrate alone were subtracted as the background.

### 4.8. Asp-Glu-Val-Asp-Ase (DEVDase) and Leu-Glu-His-Asp-Ase (LEHDase) Activity Assays

The DEVDase and LEHDase assays were performed as described in our previous studies [20]. After treatment with TRAIL in the presence or absence of volasertib, lysates were incubated with 100 μL of reaction buffer (1% NP-40, 20 mM Tris-HCl, pH 7.5, 137 mM NaCl, 10% glycerol) containing a caspase substrate [Asp-Glu-Val-Asp-chromophore-p-nitroanilide (DEVD-pNA) or Leu-Glu-His-Asp-chromophore-p-nitroanilide (LEHDase-pNA)] at 5 μM. Thereafter, the absorbance at 405 nm was measured with a spectrophotometer.

### 4.9. Quantitative PCR (qPCR)

Total RNA was isolated using the TriZol reagent (Life Technologies; Gaithersburg, MD, USA), and the cDNA was prepared using M-MLV reverse transcriptase (Gibco-BRL; Gaithersburg, MD, USA) according to the manufacturers’ instructions. For qPCR, cDNA, and forward/reverse primers (200 nM) were added to 2× KAPA SYBR Fast master mix, and reactions were performed on LightCycler 480 real-time amplification instrument (Roche, Basel, Switzerland). The following primers were used for the amplification of human c-FLIP and actin: c-FLIP (forward) 5′-CGC TCA ACA AGA ACC AGT G-3′ and (reverse) 5′-AGG GAA GTG AAG GTG TCT C-3′ and actin (forward) 5′-CTA CAA TGA GCT GCG TGT G-3′ and (reverse) 5′-TGG GGT GTT GAA GGT CTC-3′. Threshold cycle number (*C*_t_) of each gene was calculated, and actin was used as reference genes. Delta-delta *C*_t_ values of genes were presented as a relative fold induction.

### 4.10. c-FLIP Constructs and Stable Cells

The human c-FLIP expression vector was constructed, as described previously [28].

### 4.11. Statistical Analysis

The data were analyzed using a one-way ANOVA and post-hoc comparisons (Student–Newman–Keuls) using the Statistical Package for Social Sciences 22.0 software (SPSS Inc., Chicago, IL, USA).

## 5. Conclusions

Volasertib sensitizes TRAIL-mediated apoptosis in various cancer cells but not normal cells. Furthermore, downregulation of c-FLIP is involved in volasertib plus TRAIL-induced apoptosis. Therefore, we provide that volasertib could be a sensitizer of TRAIL in cancer cells.

## Abbreviations

TRAIL	Tumor necrosis factor-related apoptosis-inducing ligand
ROS	Reactive oxygen species
HSF	Human skin fibroblast
DR	Death receptor
